# Progesterone therapy for prevention of recurrent spontaneous preterm birth in a minority patient population: a retrospective study

**DOI:** 10.1186/s12884-024-06471-6

**Published:** 2024-04-08

**Authors:** Genevieve R. Mazza, Emi Komatsu, Madeline Ponzio, Claire Bai, Victoria K. Cortessis, Elizabeth B. Sasso

**Affiliations:** 1 Department of Obstetrics and Gynecology, Los Angeles County + University of Southern California (LAC+USC) Medical Center, Keck School of Medicine of University of Southern California, 1200 N State Street, Los Angeles, CA 90033 USA; 2 Keck School of Medicine of University of Southern California, Department of Population and Public Health Sciences, Los Angeles, CA USA; 3 Division of Maternal-Fetal Medicine, Department of Obstetrics and Gynecology, University of Southern California, Los Angeles, CA USA

**Keywords:** IM 17-alpha hydroxyprogesterone caproate, Vaginal progesterone, Spontaneous preterm delivery, Prematurity

## Abstract

**Background:**

Preterm birth is a leading cause of infant morbidity and mortality worldwide. The burden of prematurity underscores the need for effective risk reduction strategies. The purpose of this study is to evaluate the efficacy of progesterone therapy, both intramuscular 17-α-hydroxyprogesterone caproate (IM 17-OHPC) and vaginal progesterone, in the prevention of recurrent spontaneous preterm birth (sPTB). The co-primary outcomes included: recurrent spontaneous PTB < 37 and < 34 weeks’ gestation.

**Methods:**

This retrospective cohort study included 637 pregnant patients that delivered at any of the three hospitals within the Los Angeles County healthcare system between October 2015 and June 2021. We compared frequencies of measured variables between each of the progesterone treated groups to no treatment using Pearson chi-squared tests and independent t-tests for categorical and continuous variables, respectively. We estimated crude and adjusted associations between each specific treatment (versus no treatment) and primary outcomes using logistic regression.

**Results:**

Recurrent sPTB < 37 weeks’ gestation occurred in 22.3% (*n* = 64) of those in the no treatment group, 29.1% (*n* = 86, *p* = .077) in the 17-OHPC group, and 14.3% (*n* = 6, *p* = 0.325) in the vaginal progesterone group. Recurrent sPTB < 34 weeks’ gestation was 6.6% (*n* = 19) in the no treatment group, 11.8% (*n* = 35, *p* = .043) in the 17-OHPC group, and 7.1% (*n* = 3, *p* = 1) in the vaginal progesterone group. Among all participants, neither 17-OHPC nor vaginal progesterone was significantly associated with a reduction in recurrent sPTB at any time point. Among those with a short cervix, IM 17-OHPC was positively associated with recurrent sPTB < 37 weeks’ gestation (aOR 5.61; 95% CI 1.16, 42.9).

**Conclusions:**

Progesterone therapy of any type did not reduce the risk of recurrent sPTB < 34 or < 37 weeks’ gestation compared to no progesterone therapy.

## Background

Preterm birth (PTB) is a significant challenge to human health worldwide and remains a leading cause of infant mortality in the United States [1–3]. Prematurity increases the risk of death from other causes and is associated with significant short and long-term consequences affecting nearly every organ system [2]. A recent JAMA article reported that neurodevelopmental impairment was common at 2 years of age in infants born less than 27 weeks [4]. Prematurity has been linked to adult-onset diseases such as hypertension, obesity, and diabetes. It has been postulated that the true costs of prematurity on a global level are grossly underestimated [2].

The PTB rate in the US in 2020 was 10.09%; this small decline from 10.23% in 2019 was the first decline in rate since 2014 [5]. However, provisional CDC data for the first 6 months of 2021 suggest that the PTB rate has again risen [1]. The significant burden of prematurity underscores the need for effective risk reduction strategies.

The strongest predictor of PTB is a history of spontaneous PTB (sPTB) [6]. Additional risk factors include: number of prior preterm births and gestational age at prior preterm birth [6, 7]. In a randomized controlled trial by Meis et al., weekly injections of 17 alpha-hydroxyprogesterone caproate (17-OHPC) starting at 16–20 weeks’ gestation significantly reduced the risk of preterm delivery as well as neonatal complications compared with placebo group [8]. In response to these early results, the FDA granted accelerated approval of 17-OHPC for prevention of recurrent preterm birth (rPTB), but called for a follow up confirmatory trial. The confirmatory trial by Blackwell et. al, known as PROLONG trial, demonstrated no difference between treatment groups and concluded that 17-OHPC did not decrease rPTB [9]. The FDA then withdrew accelerated the approval for 17-OHPC in 2019. In response to these developments, the Society for Maternal Fetal Medicine (SMFM) concluded that the differences between these two trials may be partially explained by differences in study population and that it is therefore reasonable to continue use of 17-OHPC for select patients [6]. Nonetheless, uncertainty regarding the true benefit of 17-OHPC make additional data imperative. Challenges to conducting informative randomized trials, including cost and sample size considerations, were met with a call for additional observational data [7].

Additionally, vaginal progesterone became an alternative treatment in 2021 when ACOG guidelines were revised to recommend either vaginal progesterone or 17-OHPC for prevention of sPTB. Our institution serves a largely minority patient population within a large, urban, safety net hospital system. The use of 17-OHPC had been widely adopted, and the use of vaginal progesterone became increasingly common during the COVID-19 pandemic. Thus, we conducted this study to evaluate the effectiveness of progesterone therapy, both 17-OHPC and vaginal progesterone, for the prevention of recurrent spontaneous PTB. We reasoned that if progesterone therapy does reduce recurrent sPTB, patients treated with progesterone therapy of either type would experience rPTB less frequently than those who received no progesterone therapy.

## Methods

**Fig. 1:**
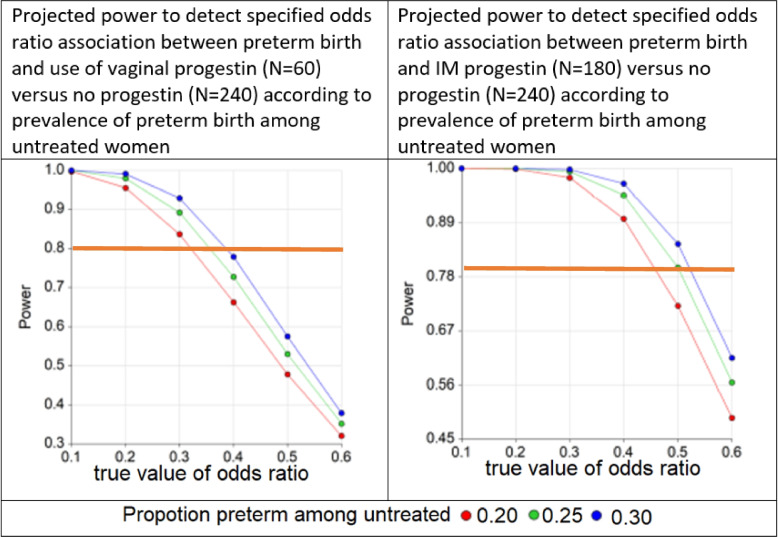
^a,b^Power curves demonstrating projected statistical power. ^a^ In the da Fonseca trial of vaginal progesterone for preterm birth, among the 72 women who received this intervention, 2 (2.8%) delivered before 34 weeks and 10 (13.9%) before 37 weeks; by comparison, among 70 who received placebo, 13 (18.6%) delivered before 34 weeks and 20 (28.5%) before 37 weeks. The corresponding odds ratio estimates were 0.13 (95% confidence interval [CI] 0.03–0.58) and 0.40 (95% CI 0.17–0.94). ^b^ In the NICHD Meis et al. trial of 17-OHPC, among 306 women receiving 17-OHPC, 35 (11.4%) delivered before 32 weeks, and 63 (20.6%) delivered before 35 weeks; by comparison, among 153 who received the placebo, 30 (19.6%) delivered before 32 weeks and 47 (30.7%) delivered before 35 weeks. The corresponding odds ratio estimates were 0.53 (95% confidence interval [CI] 0.31–0.90) and 0.58 (95% CI 0.38–0.91)

This retrospective cohort study examined medical records of obstetric patients who delivered at any of the three hospitals within the Los Angeles County healthcare system between October 2015 and June 2021. We included patients with a singleton gestation who had a history of sPTB (between 20 0/7 and 36 6/7 weeks’ gestation) in a previous pregnancy. Exclusion criteria included: known fetal anomaly, history indicated cerclage, or multifetal gestation in index pregnancy. A detailed power analysis was conducted prior to data collection, and power curves were developed providing the projected statistical power (Fig. 1). To do this, we anticipated that among approximately 1500 women per year who delivered within our hospital system over the study period (9000 total), there were 480 (80 per year) who had a previous preterm delivery, of whom 240 (40 per year) were not treated with progesterone, 180 (40 per year for 4.5 years before the COVID-19 pandemic) were treated with IM progesterone, and 60 (40 per year for 1.5 years during the COVID-19 pandemic) were treated with vaginal progesterone. Our specific institution switched from using IM progesterone to vaginal progesterone during the COVID-19 pandemic to limit exposure to healthcare settings during this time. With this anticipated distribution of exposure history, we used the normal approximation method for unequal sample sizes to estimate the statistical power. The power curves in Fig. 1 show the resulting projected statistical power to detect odds ratios of 0.1 to 0.6, which encompass the range of estimates of efficacy previously reported by Meis et al. [8] and da Fonseca et al. [10] for 3 values of preterm birth proportion among untreated participants (20%, 25%, and 30%). Based on the resulting curves, we anticipated the power of the study to likely exceed 80% to detect effect sizes of < 0.4 for vaginal progesterone and < 0.5 for IM progesterone.

Demographic information, elements of reproductive history, use of progesterone therapy, risk factors for rPTB, and patient and pregnancy outcomes were recorded. The intervention of interest, progesterone therapy, included: 17-OHPC, vaginal progesterone, or no treatment. Usual regimens at our institutions were 250 mg weekly IM 17-OHPC and 200 mg progesterone via vaginal suppository. Primary outcomes were: recurrent sPTB at each of two timepoints, < 37 weeks’ gestation and < 34 weeks’ gestation. Secondary outcomes included: first or second trimester loss, PPROM (preterm prelabor rupture of membranes), gestational diabetes (GDM), hypertensive disorder of pregnancy, gestational age at delivery, and maternal length of hospital stay; as well as neonatal outcomes including birthweight, 1 and 5 min Apgar scores, intrauterine fetal demise, neonatal demise, cesarean delivery, NICU admission, respiratory distress syndrome, bronchopulmonary dysplasia, grade III-IV intraventricular hemorrhage, necrotizing enterocolitis, sepsis, and length of NICU stay. A final secondary outcome was a derived variable estimating prolongation of the index pregnancy in relation to earlier PTBs (termed “latency” in the results), calculated as gestational age at delivery of index pregnancy minus gestational age at earliest prior PTB.

We also collected information on numerous covariates: total maternal weight gain, BMI, gestational age of earliest sPTB, number of prior sPTBs, chronic hypertension, pregestational diabetes, short cervix (≤ 25 mm), tobacco/alcohol use, illicit substance use (methamphetamine, cocaine, opioids, marijuana – analyzed individually), Hispanic and non-Hispanic Black race, short interpregnancy interval, infections (urinary tract infection, sexually transmitted infection, bacterial vaginosis – analyzed independently), antepartum vaginal bleeding, prior cervical surgery, history of uterine instrumentation, periodontal disease, and limited prenatal care (less than 5 prenatal visits). These covariates – in addition to demographic factors and features of reproductive history – we regarded as potential confounders of progesterone treatment-sPTB associations*.* The study protocol was approved by the Institutional Review Board (IRB) at the University of Southern California. Furthermore, the stated IRB waived the need for informed consent in the study protocol approval, because the use of such protected health information involves no more than minimal risk to the privacy of individuals and the research could not be practicably conducted without the waiver and without access to the protected health information.

We compared frequencies of measured variables between each of the progesterone treated groups to patients who received no treatment using Pearson chi-squared tests and independent t-tests for categorical and continuous variables, respectively. We estimated crude and adjusted associations between each specific treatment (versus no treatment) and primary outcomes using logistic regression. To select variables for inclusion as potential confounders in the multivariate model we conducted a series of analyses in which we added each potential confounder into the model, individually; we retained in the final model variables for which inclusion resulted in > 20% change for treatment-outcome pair examined. Those retained in the multivariate model were: gestational age of earliest sPTB (in days, continuous), short cervix (yes/no), limited prenatal care (yes/no), and maternal race (non-Hispanic Black/other). It is important to acknowledge the inclusion of maternal race in the multivariate model, as race is a social and not a biological factor. The chronic stress from structural racism has been proposed by ACOG (American College of Obstetricians and Gynecologists) as a possible explanation for the strikingly increased preterm birth rates seen in non-Hispanic Black patients [11]. We report results of crude and adjusted logistic regression analyses as point and 95% confidence interval (95% CI) estimates of the odds ratio (OR). To investigate whether having a short cervix modifies treatment-primary outcome associations we repeated these analyses within strata defined by whether short cervix was documented.

## Results

**Fig. 2:**
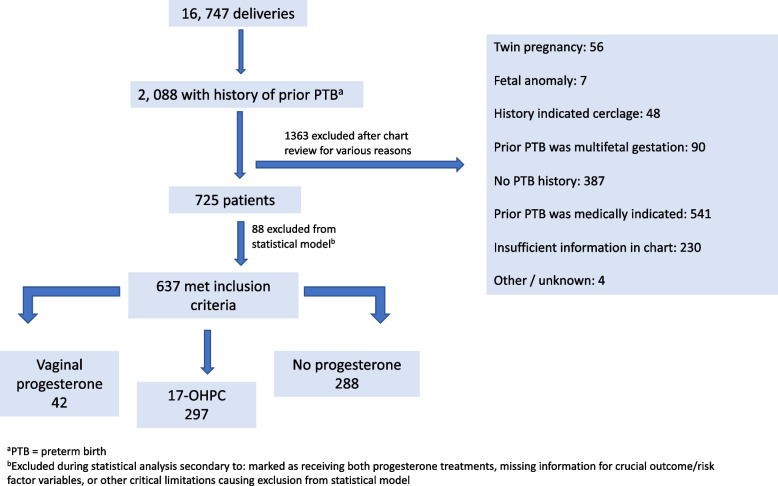
Patient recruitment

Of 16,747 deliveries during the study period, 637 women had a history of sPTB and met all inclusion criteria (Fig. 2). Demographic and clinical characteristics of the study population are found in Table 1. A total of 348 patients were started on any form of progesterone; of these 297 started on 17-OHPC and 42 on vaginal progesterone. Additionally, 288 patients received no treatment for various reasons including: provider did not recommend, patient declined, patient never initiated therapy as planned, lack of prenatal care, medical contraindication, lack of insurance coverage, or other reason. The average age of participants at the time of delivery was 30.8 ± 6.0 years. The vast majority were Latina (*n* = 460, 72.2%), followed by non-Hispanic Black (*n* = 75, 11.8%). While development of GDM is a concern in progesterone use, we observed treated groups to have only slightly elevated frequency of this condition, which did not achieve statistical significance.

**Table 1:** Demographic and risk factors for sPTB among all participants and according to specific progesterone treatment

	Total (*N* = 637)	Any Progesterone (*N* = 348)	^a^*p*-value	17-OHPC (*N* = 296)	^b^*p*-value	Vaginal Progesterone (*N* = 42)	^c^*p*-value	No treatment (*N* = 287)
**Demographic factors and reproductive history before the index pregnancy**								
Age	30.8 ± 6.0	31.0 ± 6.0	0.17	31.0 ± 6.1	0.25	30.7 ± 5.2	0.5	30.5 ± 5.9
Gravida	4 (3–5)	4 (3–5)	0.012	4 (3–5)	< .001	4 (3–5)	0.615	4 (3–6)
Parity	2 (1–3)	2 (1–3)	< .001	2 (1–2)	< .001	2 (1–3)	0.0441	2 (1–3)
^d^Race/Ethnicity			^e^0.89		^e^0.73		^e^0.49	
Non-Hispanic White	29 (4.6)	16 (4.6)		11 (3.7)		4 (9.5)		14 (4.6)
Non-Hispanic Black	75 (11.8)	41 (11.8)		34 (11.4)		6 (14.3)		34 (11.8)
Hispanic/Latina	460 (72.2)	258 (74.1)		223 (75.0)		28 (66.7)		201 (69.8)
Asian/Pacific Islander	13 (2.0)	8 (2.3)		8 (2.7)		0 (0)		5 (1.7)
Other	50 (7.8)	23 (6.6)		19 (6.4)		3 (7.1)		27 (9.4)
BMI	31.9 ± 7.1	32.1 ± 7.6	0.5	32.3 ± 7.7	0.36	30.1 ± 6.8	0.76	31.5 ± 6.4
Term delivery number	1 (0–2)	1 (0–1)	< .001	1 (0–1)	< .001	1 (0–2)	0.367	1 (0–2)
Preterm delivery number	1 (1–2)	1 (1–2)	0.829	1 (1–2)	0.798	1 (1–2)	0.808	1 (1–2)
**Demonstrated and Proposed Risk Factors for sPTB**								
Total Maternal weight gain	10.7 ± 6.4	10.4 ± 6.3	0.67	10.7 ± 6.6	0.65	9.1 ± 4.5	0.82	11.2 ± 6.7
Gestational age of earliest sPTB	32 (27–35)	32 (25–35)	< .001	32 (26–34)	< .001	32 (27–35)	0.044	34 (30–36)
Number of spontaneous preterm births 1	498 (78.2)	275 (79.0)		234 (78.8)		35 (83.3)		222 (77.1)
2	102 (16.0)	53 (15.2)		44 (14.8)		6 (14.3)		49 (17.0)
3 or more	37 (5.8)	20 (5.7)	0.89	19 (6.4)	0.89	1 (2.4)	0.93	17 (5.9)
Chronic Hypertension	53 (8.3)	29 (8.3)	0.47	26 (8.6)	0.63	3 (7.1)	0.63	24 (8.3)
Pregestational Diabetes	50 (7.8)	34 (9.8)	0.026	31 (10.4)	0.034	2 (4.8)	0.2	16 (5.6)
Short Cervix	74 (11.6)	59 (17.0)	< .001	45 (15.2)	< .001	10 (23.8)	0.001	15 (5.2)
Tobacco Use	42 (6.6)	15 (4.3)	0.055	14 (4.7)	0.054	1 (2.4)	0.89	27 (9.4)
Alcohol Use	7 (1.1)	4 (1.1)	1	3 (1.0)	1	0 (0)	1	3 (1.0)
Illicit Substance Use	67 (10.5)	32 (9.2)	0.094	29 (9.8)	0.17	3 (7.1)	0.4	35 (12.2)
Short inter-pregnancy interval	76 (11.9)	43 (12.4)	0.92	37 (12.5)	0.94	4 (9.5)	0.79	33 (11.5)
UTI	155 (24.3)	103 (27.0)	0.25	75 (25.3)	0.45	14 (33.3)	0.17	62 (21.5)
STI	60 (9.4)	32 (9.2)	0.69	26 (8.8)	0.61	6 (14.3)	0.71	28 (9.7)
BV	91 (14.3)	63 (18.1)	0.002	53 (17.8)	0.007	8 (19.0)	0.016	28 (9.7)
Antepartum vaginal bleeding	43 (6.8)	26 (7.5)	0.69	23 (7.7)	0.7	2 (4.8)	1	17 (5.9)
Prior LEEP or CKC	7 (1.1)	2 (0.6)	0.43	2 (0.7)	0.36	1 (2.4)	1	5 (1.7)
History of uterine instrumentation	93 (14.6)	58 (16.7)	0.17	48 (16.2)	0.24	8 (19.0)	0.28	35 (12.2)
Periodontal Disease	5 (0.8)	2 (0.6)	1	1 (0.3)	0.56	1 (2.4)	0.35	3 (1.0)
Limited prenatal care (< 5 visits)	83 (13.0)	11 (3.2)	< .001	8 (2.7)	< .001	2 (4.8)	0.011	72 (25.0)
Gestational Diabetes	67 (10.5)	43 (12.4)	0.26	35 (11.8)	0.42	6 (14.3)	0.28	24 (8.3)

^a^*p* value is any progesterone compared to no treatment

^b^*p* value is 17OHPC compared to no treatment

^c^*p* value is vaginal progesterone compared to no treatment

^d^16 missing race

^e^*p* value for trend

Distributions of the demographic variables differed little between treatment groups, with few notable exceptions. Several that achieved statistical significance might be expected if events in prior pregnancies influenced treatment decisions in the index pregnancy. For example, patients started on 17-OHPC had fewer prior term deliveries (0.8 ± 0.9) than those who received no progesterone (1.4 ± 1.5). Gestational age at the earliest prior sPTB was earlier in both the 17-OHPC group (29.9 ± 5.1 weeks) and the vaginal progesterone group (30.5 ± 5.4 weeks) than in those not started on progesterone therapy (32.3 ± 4.3 weeks).

Recurrent PTB at < 37 weeks’ gestation occurred in 22.3% (*n* = 64) of those in the no treatment group. By comparison, this outcome was somewhat more frequent 29.1% (n = 86) in the 17-OHPC group, and less frequent 14.3% (*n* = 6) in the vaginal progesterone group. Recurrent PTB at the earlier timepoint, < 34 weeks’ gestation, was 6.6% (*n* = 19) in the no treatment group, 11.8% (*n* = 35) in the 17-OHPC group, and 7.1% (*n* = 3) in the vaginal progesterone group.

Short cervix was documented for 74 participants, and rPTB was nearly twice as frequent among these women than other participants, OR = 1.74 (0.74, 3.78) and OR = 2.05 (1.13, 3.68) for delivery at < 34 and < 37 weeks, respectively. Specific progesterone treatment was available for 70 women with short cervix, of whom 45 started 17-OHPC, 10 started vaginal progesterone, and 15 were not treated. Owing to the anatomic basis of this risk factor we regarded short cervix as a possible modifier of associations between progesterone treatment and rPTB. We therefore report estimates for subgroups with and without short cervix, in addition to the full set of participants (Table 2).

**Table 2:** Associations between specific progesterone therapy^a^ and recurrent preterm delivery of index pregnancy at each of two timepoints

Treatment	< 34 weeks gestation		< 37 weeks gestation	
	^b^cOR (95%CI)	^c^aOR (95%CI)	cOR (95%CI)	aOR (95%CI)
Among all participants				
IM 17-OHPC	**1.89 (1.07, 3.45)**	1.79 (0.89, 3.75)	1.43 (0.98, 2.08	1.47 (0.94, 2.31)
Vaginal Progesterone	0.41 (0.25, 3.37)	1.06 (0.23, 3.62)	0.58 (0.21, 1.35)	0.59 (0.21, 1.46)
Among those without short cervix				
IM 17-OHPC	1.51 (0.79, 2.98)	1.71 (0.80, 3.84)	1.22 (0.80, 1.87)	1.36 (0.84, 2.20)
Vaginal Progesterone	0.94 (0.14, 3.53)	1.24 (0.18, 4.98)	0.54 (0.15, 1.44)	0.64 (0.18, 1.78)
Among those with short cervix				
IM 17-OHPC	1.86 (0.42, 13.1)	2.24 (0.31, 45.50)	2.88 (0.84, 11.6)	**5.61 (1.16, 42.9)**
Vaginal Progesterone	0.72 (0.03, 8.70)	1.22 (0.04, 36.73)	0.69 (0.08, 4.49)	1.45 (0.13, 16.3)

*CI* confidence interval

^a^Compared to reference group who received no treatment

^b^cOR, crude odds ratio

^c^aOR, odds ratio adjusted for gestational age at earliest prior preterm delivery, short cervix, race, limited prenatal care

### Associations between each treatment and rPTB

Table 2 provides crude and adjusted estimates of associations of each specific treatment, compared to no progesterone therapy, with the primary outcomes. Among all participants, starting 17-OHPC was positively associated with rPTB at both time points, although adjusted ORs (aOR) did not achieve statistical significance at either < 34 weeks’, aOR = 1.79 (95%CI 0.89, 3.75) or < 37 weeks’ gestation, aOR = 1.47 (95%CI 0.94, 2.31). Associations with vaginal progesterone were noticeably lower, aOR = 1.06 (95%CI 0.23, 3.62) at < 34 weeks’ and aOR = 0.59 (95%CI 0.21, 1.46) < 37 weeks’ gestation; however, both interval estimates are wide and include the null value. Corresponding estimates of these associations were similar for the large subset of women without documented short cervix. By contrast, among those with short cervix, 17-OHPC was notably more strongly associated with rPTB, aOR = 2.24 (95%CI 0.31, 45.5) at < 34 weeks’ and aOR = 5.61 (95%CI 1.16, 42.9) at < 37 weeks’ gestation, the latter statistically significant. Estimates for vaginal progesterone were particularly imprecise in those with short cervix owing to small sample size.

Frequencies of secondary outcomes in the full set of participants and within specific treatment groups can be found in Table 3. Second trimester loss occurred only in the no treatment group, and this higher frequency achieved statistical significance in comparison to 0 such losses in the 17-OHPC group (*p* = 0.011). This result was based on only 8 events and therefore may represent random error, because the analyses did not account for multiple comparisons.

**Table 3:** Frequencies of secondary outcomes of the index pregnancy in each treatment group

Secondary Outcomes	Total (*N* = 637)	Any Progesterone (*N* = 353)	^a^*p*-value	17-OHPC (*N* = 296)	^b^*p*-value	Vaginal Progesterone (*N* = 42)	^c^*p*-value	No treatment (*N* = 287)
Recurrent preterm birth (20 0/7- < 37 weeks)	161 (25.3)	99 (27.7)	0.1453	86 (29.1)	0.07666	6 (14.3)	0.3254	64 (22.3)
Recurrent preterm birth (20 0/7—< 34 weeks)	59 (9.3)	40 (11.4)	0.04774	35 (11.8)	0.04297	3 (7.1)	1	19 (6.6)
Recurrent preterm birth (20 0/7—< 28 weeks)	21 (3.3)	14 (4.0)	0.371	13 (4.4)	0.2857	0 (0.0)	0.6523	7 (2.5)
Recurrent preterm birth (20 0/7—< 24 weeks)	8 (1.3)	4 (1.1)	1	0 (0.0)	1	0 (0.0)	0.9854	8 (2.3)
Latency (days)		50.2 ± 38.97	< 0.001	49.6 ± 39.4	< 0.001	51.7 ± 37.4	< 0.001	32.98 ± 37.9
Second trimester loss (< 20 weeks)	8 (1.2)	0 (0.0)	0.005563	0 (0.0)	**0.0112**	0 (0.0)	0.5761	8 (1.2)
First trimester loss (< 14 weeks)	0 (0.0)	0 (0.0)	–	0 (0.0)	–	0 (0.0)	–	0 (0.0)
Preeclampsia	69 (10.8)	34 (9.8)	0.3722	27 (9.1)	0.2585	6 (14.3)	0.8885	35 (12.2)
Gestational age at delivery (weeks)	37.0 ± 3.9	37.0 ± 3.4	0.9542	36.9 ± 3.5	0.8699	37.9 ± 2.0	0.1856	36.9 ± 4.5
Birthweight (grams)	2991.7 ± 731.2	2956.8 ± 728.7	0.2344	2972.6 ± 723.4	0.3395	2895.4 ± 699.6	0.2601	3031.0 ± 729.7
1 min Apgar (0–9)	7.7 ± 1.9	7.7 ± 1.9	0.5895	7.6 ± 1.9	0.3538	8.2 ± 0.8	0.1332	7.8 ± 1.9
5-min Apgar (0–9)	8.5 ± 1.4	8.6 ± 1.3	0.9179	8.5 ± 1.4	0.8385	8.9 ± 0.4	0.1476	8.5 ± 1.6
Neonatal demise (demise within 30 DOL)	11 (1.7)	7 (2.0)	0.7728	7 (2.4)	0.5824	0 (0.0)	0.9831	4 (1.4)
Cesarean delivery	224 (35.3)	128 (36.9)	0.3703	110 (37.1)	0.3473	12 (28.6)	0.6509	95 (33.1)
NICU admission	181 (28.5)	108 (31.1)	0.08675	94 (31.8)	0.0715	11 (26.2)	0.9417	72 (25.1)
Respiratory Distress Syndrome	32 (5.0)	23 (3.6)	0.2602	18 (6.8)	0.2735	2 (4.8)	1	11 (3.0)
Bronchopulmonary Dysplasia	7 (1.1)	4 (0.6)	1	3 (1.0)	1	0 (0.0)	1	3 (1.0)
Grade III-IV Intraventricular hemorrhage	2 (0.3)	1 (0.2)	1	1 (0.3)	1	0 (0.0)	1	1 (0.3)
Necrotizing Enterocolitis	1 (0.2)	1 (0.2)	1	1 (0.3)	1	0 (0.0)	–	0 (0.0)
Sepsis	5 (0.8)	4 (0.6)	1	3 (1.0)	1	0 (0.0)	1	2 (0.7)
Length of NICU stay (days)	6.8 ± 18.3	7.7 ± 19.2	0.3139	8.2 ± 19.7	0.2002	2.2 ± 6.1	0.1998	5.9 ± 17.4
Length of hospital stay (days)	4.1 ± 4.0	4.3 ± 4.8	0.05204	4.2 ± 4.2	0.07702	3.9 ± 2.6	0.728	3.7 ± 2.5

^a^*p*-value compares any progesterone to no treatment

^b^*p*-value compares 17-OHPC to no treatment

^c^*p*-value compares vaginal progesterone to no treatment

Latency, defined as the difference between gestational age of earliest sPTB and gestational age at delivery of index pregnancy, was greater in treated than untreated patients. Mean latency was 49.6 days in IM 17-OHPC, 51.6 in vaginal progesterone vs 32.9 in no treatment groups (*p* < 0.001). We cannot comment on whether this difference represents a true treatment effect or confounding by indication. We also know that both progesterone groups had an earlier gestational age at earliest sPTB compared with the no treatment group, which likely contributes to the observed latency.

## Discussion

### Main findings

In the predominantly low income Latina population who participated in this research, patients who received progesterone therapy of any type did not experience significantly lower frequency of recurrent sPTB. Compared to untreated women, those who started IM 17-OHPC demonstrated greater odds of rPTB at < 34 and < 37 weeks, although multivariate estimates of the corresponding ORs did not achieve statistical significance. However, starting IM 17-OHPC was associated with five-fold greater odds of rPTB at < 37 weeks in women with documented short cervix. Use of vaginal progesterone was associated with lower odds of rPTB at < 37 weeks in women without documented short cervix, but all results for vaginal progesterone are very imprecise owing to small numbers of women who started this therapy. Thus while the data provide little information about effectiveness of vaginal progesterone in reducing the risk of recurrent sPTB in certain populations, they do indicate that IM 17-OHPC is not effective.

### Interpretation

These results reaffirm the growing body of literature that calls into question findings of the Meis trial [8]. Authors of a systematic review and meta-analysis of randomized trials of 17-OHPC for reduction of rPTB concluded that 17-OHPC may reduce the risk of rPTB at < 37 and < 35 weeks, based on data from four studies. However, this meta-analysis did not include the PROLONG data, and cannot be regarded as confirming the Meis trial because of 761 patients, 463 were the original participants in the Meis trial, and two contributing trials were not placebo controlled [12]. Thus, high quality experimental data addressing this question were available from only two studies, which reported opposing findings.

To clarify the discrepancy in the experimental data, a call was made for observational studies. Diverse populations were sought, because differing demographic makeup of the Meis and PROLONG populations allowed for the possibility that 17-OHPC may have distinct effects in different groups. One observational study had already reported rPTB to be slightly more frequent in largely Hispanic cohort of 430 women treated with 17-OHPC than in comparable patients who had received care before 17-OHPC treatment was introduced [13]. A more recent retrospective cohort study of over 800 predominantly black and high risk white women found that treatment with 17-OHPC was not associated with prolongation of pregnancy to 35 weeks’ gestation or later [14]. Thus, we report here the third relatively large observational study to contradict findings of the Meis trial, and the second of these conducted in a largely Hispanic population. Our study provides additional data challenging the effectiveness of 17-OHPC for prevention of rPTB while identifying a subgroup of high risk women – those with short cervix – for whom risk of recurrent PTB may be notably high with 17-OHPC use. Because past trials did not evaluate efficacy of 17-OHPC among women with short cervix [8, 9] or did so among few women [15], inferences about this interaction will likely rely on additional observational studies.

The small number of participants who started on vaginal progesterone provide little new information about effectiveness of this treatment. The Evaluating Progestogens for Preventing Preterm Birth International Collaborative (EPPPIC) analyses of vaginal progesterone in singleton pregnancies of women with prior sPTB provide no evidence of association with rPTB at either < 34 or < 37 weeks in women without short cervix (2 studies), and marginally significant inverse association at these time points among those with short cervix (4 studies) [15]. A meta-analysis by Romero et al. found vaginal progesterone to be associated with significantly lower risk of PTB in singleton gestations with a short cervix [16]. But their follow up meta-analysis of vaginal progesterone for prevention of rPTB in women with singleton pregnancies and history of sPTB was less convincing. This analysis identified decreased risk of rPTB at < 37 and < 34 weeks’ gestation with vaginal progesterone treatment; however, all evidence for prevention was from small trials conducted in low/middle income countries whereas large trials and those in high income countries showed no effect. The authors noted that the overall quality of evidence was poor and ultimately concluded that there was no convincing evidence supporting use of vaginal progesterone to prevent rPTB in women with a singleton gestation and a history of sPTB, particularly in the absence of a short cervix [17]. An additional prospective, observational study aimed to evaluate the association between vaginal progesterone use and prevention of recurrent preterm birth. It enrolled patients with a prior spontaneous PTB who received vaginal progesterone between 2017 and 2019 and compared outcomes to matched untreated historical controls. It found that vaginal progesterone was not associated with a reduction in recurrent PTB, and this finding was upheld regardless of prior PTB number or sequence and did not changed based on adherence [18]. Finally, the OPPTIMUM study was a double blind randomized placebo-controlled trial on vaginal progesterone use and three primary outcomes: fetal death or birth before 34 weeks 0 days (obstetric outcome), a composite neonatal outcome of death, brain injury, or bronchopulmonary dysplasia, and a standardized cognitive score at 2 years of age (childhood outcome). The researchers ultimately found that vaginal progesterone had no effect on the primary obstetric, neonatal, or fetal outcomes, thus concluding progesterone has no effect on rates of preterm birth or neonatal composite outcome. Similar to our current study, the odds ratio for the obstetric outcome was in the direction of benefit however was ultimately not statistically significant [19].

The aforementioned studies on vaginal progesterone for the primary purpose of reducing recurrent spontaneous preterm birth, particularly in the absence of short cervix, call into question the efficacy of vaginal progesterone for this sole indication. This sentiment is upheld by the findings of our study; however we acknowledge that our vaginal cohort group was underpowered to make definitive inferences. We hope that the existence of our data on vaginal progesterone can contribute to larger scale studies (ie: through meta-analyses) that aim to address this important question.

In summary, the results of our study in the broader context of the existing literature, suggest that IM 17-OHPC is not effective in reducing the risk of recurrent sPTB in a minority patient population with a history of sPTB. Providers should exercise caution when prescribing IM 17-OHPC for this indication given the accumulating data challenging its efficacy.

The greater odds of rPTB in the 17-OHPC treatment group warrants further exploration regarding whether this medication is truly harmful. One possible explanation for this finding is confounding by indication. This phenomenon, whereby patients at higher risk for recurrent sPTB may be more likely to receive treatment, could explain the more frequent rate of rPTB observed in treated participants in this and other observational studies if their higher baseline risk is not adequately addressed.

The finding of second trimester loss in our study population also warrants further investigation, initially to rule out random error as the sole explanation. Should new research validate this finding, the possibility that progesterone therapy may be just protective enough to extend pregnancy beyond 20 weeks’ gestation in select patients should also be investigated.

### Strengths and limitations

The strengths of our study include the large patient population of over 600 patients who received care under real world circumstances, and the inclusion of both IM 17-OHPC and vaginal progesterone treatment groups. Our statistical approach included detailed multivariate analyses aimed at minimizing confounding by demonstrated and suspected risk factors for preterm birth including gestational age of earliest sPTB, non-Hispanic Black race, and limited prenatal care. We also explored joint influences of progesterone therapy and short cervix. An additional strength is our investigation of a minority patient population that is often understudied.

The limitations of our study are its observational nature, retrospective capture of some data items, and the disproportionately small number of patients in the vaginal progesterone group. We endeavored to address confounding by indication, but recognize that factors that could have influenced treatment decisions may have been imperfectly measured, such that our results may reflect some residual confounding of this form. Although great care was taken to accurately and comprehensively extract all data from the medical records, the retrospective nature of the study relied heavily on accurate patient recall of medical and obstetric history and accurate provider documentation.

The authors acknowledge that the timing of the prior spontaneous preterm birth in relation to the index pregnancy affects the risk for recurrence. However, this information was not consistently available and was thus not extracted for analysis. Additionally, the earliest preterm delivery when used to calculate latency may have been remote from the index pregnancy which indeed would affect the strength of its influence in the index pregnancy outcome. Furthermore, the comparison or “no treatment” group by necessity included patients with lack of prenatal care and likely includes a heterogenous group that limits the ability to draw meaningful conclusions; however, given inclusion of this group most closely mirrors “real world” circumstances, the decision was made to include those with lack of prenatal care in the comparison group. Due to the time period of our study, the vaginal progesterone group was also relatively small and thus did not provide statistical power needed to identify any true effects of this therapy. Lastly, the authors collected data on the number of 17-OHPC injections received per subject but compliance with vaginal progesterone was not available in the dataset. Thus, the impact of compliance on outcomes was not specifically studied. While the issue of compliance likely mirrors real world adherence, it indeed could have an influential effect on the outcomes of interest. This impact of strict versus loose compliance with the treatment regimens on the results of the study cannot be understated and is thoroughly acknowledged by the authors.

## Conclusions

In women with a singleton gestation and a history of prior spontaneous PTB, treatment with progesterone therapy of any type was not associated with significantly lower frequency of recurrent sPTB at < 34 or < 37 weeks’ gestation, compared to no progesterone therapy.

## Data Availability

Data that support the findings of this study are deposited in RedCap data capture software, a HIPAA-compliant data collection software utilized by the researchers' institution. The data is stored under the project ID 7936.

## Abbreviations

IM 17-OHPC: 17-α-Hydroxyprogesterone caproate
PTB: Preterm birth
sPTB: Spontaneous preterm birth
rPTB: Recurrent preterm birth
SMFM: Society for Maternal Fetal Medicine
PPROM: Preterm prelabor rupture of membranes
GDM: Gestational diabetes
OR: Odds ratio
CI: Confidence interval
ACOG: American College of Obstetricians and Gynecologists
